# Diosgenin: Recent Highlights on Pharmacology and Analytical Methodology

**DOI:** 10.1155/2016/4156293

**Published:** 2016-12-28

**Authors:** Mafalda Jesus, Ana P. J. Martins, Eugenia Gallardo, Samuel Silvestre

**Affiliations:** ^1^CICS-UBI, Health Sciences Research Centre, Universidade da Beira Interior, Covilhã, Portugal; ^2^Laboratório de Fármaco-Toxicologia, UBIMedical, Universidade da Beira Interior, Covilhã, Portugal; ^3^Center for Neuroscience and Cell Biology (CNC), University of Coimbra, Coimbra, Portugal

## Abstract

Diosgenin, a steroidal sapogenin, occurs abundantly in plants such as* Dioscorea alata*,* Smilax China,* and* Trigonella foenum graecum*. This bioactive phytochemical not only is used as an important starting material for the preparation of several steroidal drugs in the pharmaceutical industry, but has revealed also high potential and interest in the treatment of various types of disorders such as cancer, hypercholesterolemia, inflammation, and several types of infections. Due to its pharmacological and industrial importance, several extraction and analytical procedures have been developed and applied over the years to isolate, detect, and quantify diosgenin, not only in its natural sources and pharmaceutical compositions, but also in animal matrices for pharmacodynamic, pharmacokinetic, and toxicological studies. Within these, HPLC technique coupled to different detectors is the most commonly analytical procedure described for this compound. However, other alternative methods were also published. Thus, the present review aims to provide collective information on the most recent pharmacological data on diosgenin and on the most relevant analytical techniques used to isolate, detect, and quantify this compound as well.

## 1. Introduction

**Figure 1:**
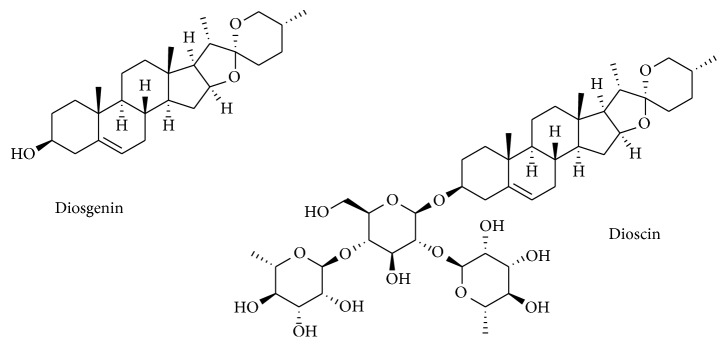
Chemical structures of diosgenin and dioscin.

The use of natural products, including steroidal compounds, has been growing not only as therapeutically active agents but also as lead compounds in drug discovery approaches [1, 2]. As a relevant example, it was discovered several years ago that a number of steroidal saponins and sapogenins share interesting anticancer properties and a relatively safe usage profile [3–5]. Amongst these compounds, diosgenin, a well-known steroidal sapogenin which originated by the hydrolysis of the saponin dioscin (Figure 1), which can be obtained from several plants, namely, from* Dioscorea*,* Trigonella, Costus* [5–7], and* Smilax* species [8], is classically used in traditional medicine against a variety of medical conditions. This steroid is of high industrial importance and has been subject of interest to many researchers worldwide over the years. In fact, most of the therapeutically useful steroidal drugs, including sex hormones and corticosteroids, are produced in a semisynthetic fashion from natural precursors and predominantly from diosgenin [9, 10]. However, in addition to this high synthetic relevance, diosgenin itself has several important biological activities also with great interest for the pharmaceutical industry [5, 7, 11]. In fact, diosgenin has been described in the literature for its pharmacological potential, including the interesting underlying mechanisms of action, thereby confirming and extending the knowledge from its usage in traditional medicine. In this context, mainly over the past two decades, a series of preclinical and mechanistic studies have been performed to understand the real importance and benefits of diosgenin against a variety of pathologies including metabolic diseases (diabetes, obesity, and dyslipidemia, including hypercholesterolemia), inflammatory diseases, and cancer [5, 7, 12]. Altogether, the results from several studies have been implicating the potential use of diosgenin as a novel multitarget based chemopreventive or therapeutic agent against several chronic ailments.

For these reasons, it is of high interest to develop efficient strategies to concentrate diosgenin from its natural sources as well as drug dosage forms to allow its administration [1, 13], either isolated or in plant extract. In addition, several pharmacokinetics studies [14] involving this compound have been performed in the last years. As such, the development of analytical methods to detect and quantify this important steroid in different matrices assumes major relevance.

Therefore, the most relevant analytical techniques used to isolate, detect, and quantify diosgenin, as well as the most recent pharmacotherapeutical data of this compound, will be presented and discussed in this review.

## 2. Pharmacology: Recent Data

Diosgenin is a steroidal sapogenin known for years for its interesting bioactivity, and accordingly a large amount of studies has been performed to explore its potential interest in a large variety of medical conditions. In fact, this compound is known to possess anti-inflammatory and antioxidant properties [15] and can be useful, for instance, in blood and cerebral disorders, allergic diseases, diabetes and obesity [16], menopausal symptoms, and skin aging; it can also have a protective role in cardiovascular diseases (such as thrombosis and atherosclerosis) [17–22] and, more importantly, in cancer [5, 11, 23–25]. In this section, a selection of the most recent discoveries on the pharmacological interest of diosgenin is presented.

### 2.1. Anticancer Activity

The development of cancer therapeutics from steroidal compounds has been an attractive choice for medicinal chemists and many active molecules have emerged [2, 26].

In this context, several preclinical studies investigated the effects of the diosgenin as a chemopreventive/therapeutic agent against cancers of several organs, and this has demonstrated the high interest of this molecule as a potential antitumor agent [5, 7]. In fact, the anticancer effect of diosgenin has been studied in various tumoural cell lines and it was evidenced that this bioactivity depends both on the cell type and on concentration. Thus, for example, diosgenin has antiproliferative activity, namely, in prostate cancer (PC-3 and DU-145 cells) [23], colon carcinoma (HCT-116 and HT-29 cells) [24], erythroleukemia (HEL cells) [27], squamous carcinoma (A431, Hep2, and RPMI 2650 cells) [28], hepatocellular carcinoma (HepG2 and HCC cells) [6, 25, 29], gastric cancer (BGC-823 cells) [30], lung cancer (A549 cells) [31], breast cancer (MCF-7) [6, 32–34], and human chronic myeloid leukemia (CML) (K562 cells) [1]. Moreover, several studies suggested that the known anticancer mechanisms of action of diosgenin are associated with a modulation of multiple cell signalling events involved in cell growth/proliferation, differentiation, epithelial-mesenchymal transition migration, and apoptosis, as well as oncogenesis and angiogenesis [12]. Within the various phases of tumorigenesis, diosgenin seems to be critical in inducing apoptotic cell death and avoiding their malignant transformation [3, 5, 12]. More specifically, the diosgenin antitumor effects have been demonstrated, for example, to be mediated through p53 activation, immune-modulation, cell cycle arrest, modulation of caspase-3 activity, and activation of the transcription STAT3 signalling pathway [6, 7, 25]. In this context, important studies have shown that diosgenin inhibits the proliferation of osteosarcoma cells by inducing apoptosis and cell cycle arrest in G1 phase [35] and also inhibits the proliferation of breast cancer cells (MCF-7 cells) through the induction of the proapoptotic p53 protein and an increase of caspase-3 levels [6, 36]. In addition, the proliferation of PC-3 human prostate cancer cells is inhibited by diosgenin in a dose-dependent manner, reducing cell migration and invasion by decreasing matrix metalloproteinase expression which reveals the potential of this compound in antimetastatic therapy [23]. Diosgenin, due to its antioxidant activity, affects the growth of A549 lung cancer cell line and downregulates hTERT gene expression in these cells in a time dependent manner. Therefore, this sapogenin could constitute an interesting approach for lung cancer therapy [31, 37]. The diosgenin-induced apoptosis of HEL cells (human erythroleukemia cell line) was related to COX-2 upregulation. In addition, this apoptosis induction was accompanied by an increase in Bax/Bcl-2 ratio, PARP cleavage, and DNA fragmentation [38]. In the COX-2 deficient K562 cells, the inhibition of NF-kappa B nuclear binding and p38 MAPK activation are involved in the diosgenin-mediated signal cascades for inducing/regulating DNA fragmentation [39]. Other authors also demonstrated that this steroid inhibits the proliferation of this leukemia cell line via cell cycle G2/M arrest and apoptosis, with disruption of Ca^2+^ homeostasis and mitochondrial dysfunction playing vital roles [40]. Moreover, diosgenin not only produces cytotoxic effect on human chronic myeloid leukemia cells (K562 and BaF_3_-WT) but also induces autophagy accompanied by reactive oxygen species (ROS) generation and mammalian target of rapamycin (mTOR) signalling pathway inhibition. Further studies also demonstrated that the inhibition of autophagy potentiated the diosgenin-induced apoptosis [1]. Diosgenin inhibits the STAT3 signalling pathway in the human hepatocellular carcinoma (HCC) cells, leading to the suppression of cell proliferation and to chemosensitization, and caused arrest at the G1 phase of the cell cycle and induced apoptosis through caspase-3 activation and PARP cleavage occurred [41]. In HepG2 hepatic cells, this steroid induces apoptosis through the Bcl-2 protein family (Bcl-2, Bax, and bid) mediated by the mitochondrial/caspase 3-dependent pathway. Furthermore, diosgenin also generates ROS and leads to oxidative stress which might induce apoptosis [25]. Furthermore, the colorectal adenocarcinoma cell line HT-29 is sensitized by diosgenin to TRAIL (TNF-related apoptosis-inducing ligand) induced apoptosis [24].

Diosgenin also has antimetastatic effects; for example, it was demonstrated that it can inhibit the migration of human breast cancer MDA-MB-231 cells, at least partially, by suppressing Vav2 protein activity [42]. Additionally, angiogenesis is an essential process for the development, invasiveness, and metastasis of solid tumours and is dependent on the action of angiogenic factors, namely, integrin and VEGF. In this context, it has been reported that VEGF expression in PC-3 cells is reduced by diosgenin in a dose-dependent manner, suggesting that this steroid can inhibit angiogenesis by interfering with this factor [23]. All of these results have shown significantly the potential use of this compound as a new therapeutic agent against various types of cancer. Thus, there has been considerable effort to continue assessing the role of diosgenin and some of its chemical analogues as well as combinations of diosgenin with other bioactive compounds in modulating growth and proliferation of various types of human tumours and in the evaluation of its potential mechanism of action. As a relevant example, the combination of diosgenin and thymoquinone has antiproliferative and apoptotic effects on squamous cell carcinoma (SCC), in a synergistically way, and thus could be a novel strategy for the development of potential antineoplastic therapies against squamous cell carcinoma [28].

An interesting novelty in this topic is the integration of diosgenin, as well as other interesting potential drugs, into nanoparticles, in order to drive diosgenin to its site of action and to increase its pharmacological bioavailability. In fact, diosgenin functionalized iron oxide nanoparticles, as well as hollow manganese ferrite nanocarriers encapsulating tamoxifen and diosgenin, were developed as potential therapeutic tools against breast cancer [34, 43]. Also in this context, Li et al. [44] prepared, characterized, and evaluated a nanoparticle platform based on poly(ethylene glycol)diosgenin conjugates for codelivery of anticancer drugs as a promising drug delivery system for cancer therapy.

### 2.2. Anti-Inflammatory and Immunological Activity

The anti-inflammatory activity of diosgenin is a known relevant effect of this steroid and has relevant interest in a variety of pathologies; however, its mechanism of action is still unclear. In this context, Jung et al. [45] observed a reduction in the production of several inflammatory mediators, including NO and interleukins 1 and 6, in murine macrophages which had been pretreated with diosgenin and stimulated with lipopolysaccharide/interferon-*γ*. In addition, the inhibitory effect of diosgenin on superoxide generation was investigated in bone marrow activated neutrophils (in the mouse) and it was evidenced that this steroid potently and concentration-dependently inhibited the extracellular and intracellular superoxide anion generation. Moreover, this effect was associated with a blockade of cAMP, PKA, cPLA 2, PAK, Akt, and MAPKs signalling pathways [46].

As atherosclerosis is a chronic inflammatory disease whose progression depends on the expression of adhesion molecules on vascular smooth muscle (VSMC) cells, the anti-inflammatory activity of diosgenin in this condition was also studied. In this study, it was observed that this steroid reduced the adhesive capacity of VSMC cells and the TNF-*α* mediated induction of ICAM-1 and VCAM-1 in VSMC by inhibiting the MAPK/Akt/NF-*κ*B signalling pathway and ROS production [17]. This explains the ability of this compound to suppress inflammation within the atherosclerotic lesion and to modulate the immune response. Very recently, it was evidenced that diosgenin regulates adipokine expression in perivascular adipose tissue and ameliorates endothelial dysfunction via regulation of AMPK which can also explain its capability to protect endothelial functions against inflammatory insults [47].

In addition, the effect of diosgenin on modulating food allergy was investigated in BALB/c mice and a suppressive effect on the intestinal inflammation was evidenced, including the occurrence of diarrhoea, the infiltration and degranulation of mast cells, and the presence of mucin containing goblet cells in the duodenum. In addition, it was demonstrated that the in vivo antiallergic activity of diosgenin is associated with the suppression of IgE production and mast cell infiltration and degranulation [48].

A recent study demonstrated that the administration of diosgenin provides a significant protection against the monocrotaline-induced pulmonary hypertension in rats. In fact, diosgenin treatment preserved hemodynamic changes and alleviated oxidative stress, inflammatory, and apoptotic markers induced by monocrotaline. This protective effect could be mediated through preserving eNOS expression together with inhibition of iNOS overexpression [49].

In addition, the suppressive effects of long-term diosgenin treatment on phthalic anhydride-induced skin inflammation using IL-4/Luc/CNS-1 transgenic mice with luciferase cDNA regulated by human IL-4 promoter and enhancer of IL-4 (CNS-1) was also evaluated. The results verified the correlation of IL-4 with suppression of this steroid in skin inflammation induced by repeated dermal exposure to phthalic anhydride [50].

As osteoarthritis is characterized by progressive destruction of articular cartilage and synovial inflammation, diosgenin can also be of interest in this disease due to its anti-inflammatory and immunomodulating properties. In fact, it was demonstrated that this steroid inhibits IL-1*β*-induced expression of inflammatory mediators, including metalloproteinases 3 and 13, inducible nitric oxide synthase, and COX-2 in human osteoarthritis chondrocytes [51]. In this context, it was also demonstrated that diosgenin increased the expression of VEGF, angiopoietin, and endothelial tyrosine kinase receptor and therefore can be a molecule of interest in rheumatoid arthritis [52].

### 2.3. Anti-Infectious Activity

Diosgenin was also investigated for its anti-infectious effects, namely, against fungi, bacteria, protozoa, and virus. Concerning the human pathogenic yeasts* Candida albicans*,* C. glabrata,* and* C. tropicalis* it was found that this steroid has weak antimicrobial activity against all the tested organisms [53, 54]. In addition, diosgenin also has low to null effect against the fungi* Aspergillus flavus*,* Aspergillus niger, Trichoderma harzianum,* and* Fusarium oxysporum*. On the other hand, this sapogenin exhibited significant susceptibility against various Gram-positive (*Bacillus subtilis*,* Bacillus cereus*,* Staphylococcus aureus,* and* Staphylococcus epidermidis*) and Gram-negative (*Escherichia coli* and* Salmonella typhi*) pathogens [55]. In addition, the antiamebic activity of diosgenin against* Naegleria fowleri* trophozoites at the cellular and molecular levels was also investigated. Interestingly, it was suggested that diosgenin has activity against the surface membrane and the* nf cysteine protease* of* N. fowleri* trophozoites. Moreover, the toxicity to mammalian cells caused by this steroid at therapeutic levels was lower than that of amphotericin B, the drug used currently to treat* N. fowleri* infections [56]. Furthermore, diosgenin revealed to be an interesting molecule in some viral diseases. In fact, due to its antioxidant activity, diosgenin can be useful in HIV patients with dementia [57]. In addition, this steroid exhibits antiviral activity against Hepatitis C Virus (HCV) in in vitro studies. Since diosgenin can reduce plasma cholesterol and HCV requires cholesterol for an efficient replication, this effect can be associated with the inhibition of viral replication [58].

### 2.4. Effects in Diabetes, Dyslipidemias, and Obesity

Concerning other relevant biological activities, according to several in vitro and in vivo studies, this phytosteroid possesses protective benefits against metabolic diseases such as diabetes and obesity [16, 19, 59, 60], metabolic syndrome [61], and dyslipidemias, including hypercholesterolemia [7, 21, 62, 63]

In fact, diosgenin can be useful in the treatment of diabetes by promoting adipocyte differentiation and by inhibiting inflammation in adipose tissues. Therefore, diosgenin may be useful to improve the patient's condition in the glucose metabolic disorder associated with obesity [64]. In this context, in other experimental models, it was observed that diosgenin led to a reduction of plasma and hepatic triglycerides in obese diabetic mice and may be useful for the management of diabetes-related hepatic dyslipidemias [65]. Furthermore, in diosgenin-treated diabetic rats a reduction of hyperglycemia, hypercholesterolemia, and hypertriglyceridemia was observed, as well as improved levels of the antioxidant enzymes SOD and GPx and a minimized level of lipid peroxidation. The adipogenic activity of diosgenin was influenced by PPAR *γ* and PPAR *α* [59]. Furthermore, the antiatherogenic effects of this steroid can be explained not only by a reduction on intestinal cholesterol absorption but also via suppression of the MiR-19b induced downregulation of ATP-binding cassette transporter A1 in macrophages [66].

This compound also has a positive effect on the endothelial dysfunction associated with insulin resistance by means of an IKK*β*/IRS-1-dependent manner and therefore can be useful in the prevention or treatment of cardiovascular disorders involved in insulin resistance and diabetes [19]. Later it was demonstrated that chronic administration of diosgenin to diabetic rats has a hypoglycemic effect and could restore vascular reactivity via endothelium-dependent and independent mechanisms and at least partially by offsetting lipid peroxidation, apoptosis, and inflammation [16].

In another study it was demonstrated that, after administration of diosgenin to diabetic rats, the activity of glucokinase decreased, while the activities of glucose-6-phosphatase and fructose-1,6-bisphosphatase in the liver have increased. Furthermore, amongst other positive changes in several parameters associated with diabetes, the supplementation with diosgenin decreased blood glucose levels in diabetic rats when compared to the group of rats fed with normal diet. This result correlates with the previous reports stating that diosgenin has hypoglycemic properties [21]. In this context, other relevant enzymes in diabetes were modulated by diosgenin [67, 68].

Interestingly, it was evidenced that fenugreek seed extracts have hepatoprotective effects which could be associated with diosgenin acting through attenuation of endoplasmic reticulum stress and oxidative stress in type 2 diabetic rats [69].

As diabetes can also lead to important changes in renal function, several studies were conducted in experimental models to evaluate this situation. For example, in a study on renal tubular fibrosis it was demonstrated that diosgenin, because of its anti-inflammatory effects, also played a protective role against high glucose-induced renal tubular fibrosis possibly by means of the epithelial-to-mesenchymal transition (EMT) pathway [70]. Also, the effectiveness of diosgenin as an antioxidant agent was evident, for example, from its effect on the renal antioxidant system and oxidative markers such as myeloperoxidase and lipid peroxidation. Therefore, diosgenin exhibited a protective effect on the kidney in diabetic rats, implying that it could be a potential candidate for treatment of diabetes with renal associated complications [60].

### 2.5. Anticoagulant and Antithrombotic Effects

In in vitro and in vivo models it was demonstrated that diosgenin exerts antithrombotic activity via inhibition of platelet aggregation and thrombosis and by prolonging APTT, PT, and TT in rats in a dose-dependent manner. This compound also prolonged bleeding and clotting times and increased protection rate in mice, again in a dose-dependent manner [22, 71]. In addition, more recently it was again demonstrated that this steroid and a structural analogue act by inhibiting platelet aggregation, which prevents blood coagulation [72]. Due to this interesting effect, an amphiphilic supramolecular prodrug consisting of a diosgenin derivative (theophylline diosgenin) and uracil terminated poly(ethylene glycol) was developed to enhance drug solubility and to prolong its systemic circulation. Interestingly, not only was a better antithrombotic activity and platelet aggregation compared to diosgenin observed, but this system had also low toxicity [73].

### 2.6. Others

Another important effect of the antioxidant diosgenin is its potential interest in the protection of cardiac cells from hypoxia-reoxygenation injury which can be mediated by ATP-sensitive potassium channels and through modulation of cell prodeath (Bax) and cell prosurvival (Bcl2, heme oxygenase 1, and Akt) molecules [74–78].

Recent studies indicated that diosgenin may protect against bone loss, namely, in experimental models of senescence, menopause, and retinoic acid-induced osteoporosis [79–81]. However the mechanism of action is still not clear but can be associated with a modulation on the receptor activator of NF-kB ligand/osteoprotegerin ratio [80].

The effects of diosgenin in a mouse model of Graves' disease were also investigated and it was observed that this steroid can relieve goiter through the inhibition of thyrocyte proliferation. In addition, the mechanisms for this action involve the suppression of IGF-1, NF-*κ*B, cyclin D1, and PCNA expression [82].

As a natural antioxidant, diosgenin is known to have neuroprotective effects and to improve some aging-related deficits, namely, memory improvement. Thus this steroid has potential interest in neuropathies such as neurodegenerative diseases, including Alzheimer's disease [5]. In this context, it was demonstrated recently that the diosgenin-induced cognitive enhancement in normal mice neurons is mediated by the membrane-associated rapid response steroid-binding receptor (1,25D_3_-MARRS) [83]. In another study, the neuroprotective potential of diosgenin in a pentylenetetrazole induced kindling model of epilepsy in mice was demonstrated. In spite of an improvement of the oxidative markers which was observed, the mechanism of this diosgenin action remains unknown [84].

## 3. Analytical Methods

Due to the extensive range of pharmacological properties, the detection and quantification of saponins [85] and diosgenin in different matrices became imperative. This is also important to the study of the pharmacokinetic and pharmacodynamic properties of this steroid and to the development of pharmaceutical formulations containing it.

In this context, there are several analytical methods described in the literature for the detection and quantification of diosgenin and the most relevant of these are described in this section.

One of the first points that must be taken into account is the matrix from which diosgenin has to be extracted. As it is present in several medicinal plants [5, 7], these constitute the matrix most usually used in extraction processes for further analysis. However, other matrices can be involved too, including cosmeceutical/pharmaceutical herbal formulations, plant cell cultures, and also rat plasma samples [86–89]. However, it is important to highlight the difficulty in detecting this type of sapogenins in biological fluids due to their low concentration and lack of a chromophore in the molecule [86]. At the moment, to the best of our knowledge, there are still no validated analytical methods for the detection of diosgenin in human biological samples.

### 3.1. Plant Matrices and Herbal Formulations

#### 3.1.1. Extraction Procedures

Several research studies concerning the isolation and purification of diosgenin, mainly from plants, have been performed. In general, direct acid hydrolysis of dioscin and spontaneous fermentation or enzymatic catalysis followed by liquid-liquid extraction (LLE) or solid phase extraction (SPE) are the most commonly used techniques to obtain diosgenin. However, these procedures can have disadvantages such as low efficiency, need of high volumes of solvents, and contamination of the extract with potentially toxic solvents and sometimes long extraction times are needed [90]. For these reasons, other methods have been developed to extract diosgenin, namely, from* Rhizoma dioscoreae*, involving supercritical fluid extraction (SFE) (using supercritical CO_2_) after acid hydrolysis, followed by high-speed counter-current chromatography (HSCCC) with evaporative light scattering detection (ELSD) [91]. In addition, the previously referred to conventional techniques were optimized using multienzymatic catalysis in combination with acid hydrolysis, allowing obtaining high purity diosgenin (>96%) from* Dioscorea zingiberensis C. H. Wright* [90]. Moreover, a focused microwave-assisted extraction (MAE) followed by acid hydrolysis was developed by Kaufmann et al. [92] to extract diosgenin from fenugreek* (Trigonella foenum graecum)* leaves and roots. The preparation of plasma samples for further analysis can be achieved through a single-step procedure of protein precipitation (PPT) [86].

#### 3.1.2. Analytical Methods

Classical analytical methods [93] for the detection/quantification of diosgenin included techniques such as spectrophotometry, gravimetry, and thin-layer chromatography (TLC). For example, through TLC the characterization of diosgenin from extracts of in vitro cultured tissues of* Helicteres isora Linn*. was also possible [94]. In addition, as classical techniques presented some drawbacks, other methods have emerged, in particular, more advanced TLC methods (e.g., HPTLC), immunoenzymatic assays (ELISA), GC, LC, UPLC, UHPLC, and HPLC, coupled to different detectors. Nuclear magnetic resonance spectroscopy is another analytical technique which can be very useful in the detection and characterization of diosgenin and other sapogenins isolated from plants [95, 96].

As a relevant example, an optimized and validated method involving TLC that overcomes the background interference problems in postderivatization was described by Trivedi et al. [97]. This was achieved through the use of a modified anisaldehyde-sulfuric acid reagent which allowed visualizing the spots and the quantification of diosgenin was performed by densitometry. Later, a validated TLC method for the simultaneous detection and quantification of diosgenin and sarsasapogenin in* Asparagus officinalis* L. was developed. In this method the plant extract was acid-hydrolyzed and after a liquid-liquid extraction a densitometric-TLC was performed. The results were verified by HPLC-UV and HPLC-MS [98].

HPTLC is an advanced form of TLC, having different enhancements aiming to increase the resolution of the compounds to be separated (e.g., using finer particle sizes in the stationary phase and/or multiple developments of the plate) and to allow their quantitative analysis by different detection/quantification systems (e.g., UV, diode array, and mass spectrometry) [99]. Due to its advantages, this technique was also successfully applied to detect and quantify diosgenin in different matrices. In this context and as an example, Nagore et al. [100] developed simple, rapid, accurate methods using HPTLC and HPLC for the determination of diosgenin in fenugreek seeds. Both methods were precise and specific and there was no statistical significant difference between them. Other HPTLC methods have been validated for the determination of diosgenin also in fenugreek seeds and in marketed formulations [101, 102]. In addition, by means of the HPTLC technique, the diosgenin content in fifteen different* Trigonella* species was determined, including seeds and aerial parts of the plant, and it can be concluded that the seeds of the species* Trigonella foenum graecum* present the highest diosgenin level [103]. Interestingly, several authors also used this technique for the quantification of diosgenin from Ayurvedic polyherbal formulations. For example, Keshwar et al. [104] developed and validated an HPTLC technique for the determination of diosgenin in a polyherbal tablet containing* Tribulus terrestris*. In this type of formulations there are other ingredients and excipients that can cause interferences in the analytical processes and thus it is of major interest to develop methods to overcome this problem. Featuring a linearity range of 240–1440 ng, this method proved to be simple and fast for routine quality control analysis of diosgenin without interference from other ingredients, excipients, or auxiliary substances. Another similar method involving densitometric HPTLC was developed and validated by Parameswaran and Koshti [105], showing a linearity range for diosgenin from 1.0 to 3.0 *μ*g^−1^ per spot. This study allowed the quantification of diosgenin not only from* Gokshuradi guggulu* but also from two Ayurvedic formulations containing it.

GC-MS methods are also described in the literature. An important work in this context was performed by Taylor et al. [106] which described the analysis of steroidal sapogenins from Amber Fenugreek* (Trigonella foenum graecum)* by capillary GC and combined GC-MS. Interestingly, diosgenin was the main compound detected in seed and foliage extracts hydrolyzed with hydrochloric acid. Later, the same research group used capillary GC to study and improve the conditions for extraction of steroidal saponins with various alcohols and the conditions for subsequent hydrolysis of the isolates with sulfuric acid mixtures, using defatted seed material from Amber Fenugreek [107]. The application of this method was successfully used to study the variation in diosgenin levels in 10 accessions of fenugreek seeds produced in western Canada to assess whether genetic (accession) and environmental factors (site and year of production) influenced levels of diosgenin [108]. As another relevant example, Kaufmann et al. [92] described an analytical method for the detection of diosgenin in different plant parts (seeds, air-dried roots, and both air-dried and fresh leaves) of fenugreek* (Trigonella foenum graecum)* using a microwave-assisted extraction and capillary GC-MS. More recently, de Lourdes Contreras-Pacheco et al. [109] determined diosgenin contents by GC-MS in a tuber collection of* Dioscorea* spp. in the state of Jalisco, Mexico.

As a technique for universal quantification for routine analysis in laboratory, it is not surprising that HPLC is probably the most used for the quantification of this compound [110]. In this context, Table 1 summarizes different matrices as well as different chromatographic conditions and detection methods used for the quantification of diosgenin based on the HPLC technique. Clearly the most common matrices are, in fact, different plant species, mainly belonging to the* Dioscorea*,* Smilax, Trigonella, *and* Tribulus* genus, from which diosgenin can be extracted. Other matrices include pharmaceutical forms (e.g., tablets, capsules) of herbal formulations in which extracts of these species of plants are present.

Concerning detection, it is known that it is possible to combine HPLC with different detection techniques and that mass spectrometry (MS) and photodiode array (PDA) detection ensure better information, as well as a rapid quantitative and qualitative analysis of the constituents in plant extracts and herbal products [111].

In this context, several HPLC methods have been developed and validated for the analysis and determination of sapogenins in several matrices including, for example, HPLC-ESI/MS, used in the kernel cake of* Balanites aegyptiaca* [112] and HPLC-ELSD-UV, involved in the analysis of species of* Dioscorea* spp. [113]. The quantification of diosgenin by HPLC-DAD-UV has also been accomplished in a* Dioscorea polygonoides* tuber collection from Colombian Flora [114]. Recently, an HPLC-UV method was applied to quantify diosgenin in aqueous extracts of fenugreek seeds aiming to support the preparation of the extracts and to standardize the diosgenin levels for further use in studies of the diosgenin potential antifertility effects [115].

This technique was also applied to support the development of methods to obtain diosgenin from dioscin present in natural sources. For example, Yang et al. [116] developed and validated a method for the quantitative analysis of diosgenin in* Rhizoma Dioscorea zingiberensis *including a new approach to hydrolyze dioscin avoiding conventional methods that involve a long period of exposure of the plant to a strong acid. For this, the cellulase enzyme promoted the release of dioscin from plant cells through the breaking of *β*-D-glycoside bonds of cellulose followed by a two-phase acid hydrolysis to supplement the diosgenin extraction. Then, RP-HPLC-UV allowed the analysis of diosgenin of the prepared sample [116]. Recently, in a study aiming to purify and characterize a glycosidase obtained from a* Gibberella intermedia* WX12 strain the HPLC technique was used to determine the conversion of dioscin from* Dioscorea zingiberensis* C. H. Wright to diosgenin by means of this enzyme [124].

A technique using high-speed counter-current chromatography (HSCCC) in combination with ELSD was developed for the isolation and separation of chemical compounds in crude extracts obtained after supercritical fluid extraction and acid hydrolysis from* Rhizoma dioscoreae*, a common plant used in traditional Chinese medicine. The purities of the products were determined by HPLC and their chemical structures were identified by MS, UV, and comparison with standards [91].

Li et al. [93] described two methods to quantify diosgenin in* Dioscorea zingiberensis* cell cultures by using HPLC coupled to photodiode array detector or with a microplate spectrophotometry technique. The LOD and LOQ values presented for the HPLC technique (resp., 0.0372 *μ*g and 0.1127 *μ*g) are significantly lower than those achieved with the microplate spectrophotometry technique (resp., 0.6111 *μ*g and 1.8518 *μ*g). Therefore, higher sensitivity was possible with the HPLC technique; however, the spectrophotometry results were in good agreement with those obtained by HPLC [93]. More recently, Deshpande and Bhalsing [94] also isolated, characterized, and quantified diosgenin obtained from in vitro cultured tissues of* Helicteres isora Linn*. and plant parts. The analytical techniques used in this work were TLC, Fourier transform infrared spectroscopy (FTIR), and HPLC-UV for diosgenin characterization and spectrophotometry for quantification. In this study it was proposed that* Helicteres isora Linn.* can be an alternative source of sapogenins, including diosgenin, and it was evidenced that the amount of diosgenin obtained from in vitro cultured cells is higher than that isolated from parts of the plant [94].

In order to increase specificity and precision and to reduce the analysis time and solvent consumption, the UPLC technique coupled to different detectors was also applied to detect and quantify diosgenin. For example, an UPLC-DAD-MS was developed and validated for the identification and determination of diosgenin in several plants, and the presence of this steroid in three* Dioscorea* species and one species of* Heterosmilax* was demonstrated. In addition, it was also suggested that* D. zingiberensis* can be an important diosgenin source [117]. A UHPLC-based technique with evaporative light scattering detection (ELSD) was developed and validated for the determination of eleven steroidal saponins and diosgenin from several species of* Dioscorea*. The confirmation of the identity of these compounds was achieved through UHPLC-MS with a quadrupole mass analyzer and an ESI source [123].

In addition, there are several studies reporting the quantification of diosgenin in medicinal plants by means of LC techniques coupled to different detection systems, mainly MS. For example, the characterization of steroidal saponins in* Helleborus niger L*. roots and sapogenin products of their fermentative transformation were performed by means of LC-MS^n^ [125]. The characterization of metabolite-saponins from fruit mesocarp, kernel, and root of* Balanites aegyptiaca* was also achieved through the use of LC-ESI/MS and matrix assisted laser desorption/ionization-time of flight-mass spectrometry (MALDI-TOF/MS). Interestingly, from methanolic extracts of these plant parts 24 different saponins have been found as well as diosgenin, which was found to be the sole aglycone form present [126]. In addition, the qualitative and quantitative analysis of diosgenin obtained from* Aspergillus oryzae*-mediated biotransformation of* Dioscorea zingiberensis *raw herb saponins was described by Qi et al. [127]. In this study diosgenin quantification was performed by LC-UV, while its identification was made by the ELSD method.

More recently, a microwave-assisted extraction and a new determination method for total steroid saponins from* Dioscorea zingiberensis* C. H. Wright was optimized, validated, and compared to other conventional extraction processes. Diosgenin was quantified by HPLC-DAD and examined further by LC-ESI/MS after acid hydrolysis [128].

Immunoenzymatic tests, including ELISA, can also be a potential tool for the analysis of natural products in complex matrices, including medicinal plants. In this field, Li et al. [129] developed an indirect competitive ELISA method to quantify diosgenin, namely, in* Paris* and* Dioscorea* species. The diosgenin molecule, which is too small to be considered an immunogen, was conjugated with bovine serum albumin (BSA) to create the immunization. Then, from rabbits, a specific polyclonal antibody was developed against diosgenin-BSA conjugate. This method allowed a screening of several Chinese plants which have diosgenin as component [129]. A similar strategy was also developed to detect and quantify sarsasapogenin, a steroidal sapogenin structurally similar to diosgenin. However, a minor cross reactivity was observed, namely, to diosgenin [88].

In 2014, a new certified reference material for diosgenin using mass balance approach and coulometric titration (CT) method was developed and can be an important tool for the validation of analytical methods. Thus, diosgenin has been selected as a candidate reference material (CRM) for which the characterization was based on two different methods, mass balance and CT. In addition, an HPLC technique coupled with a diode array detector was developed and validated to be used as confirmation of the two previously mentioned methods. Therefore, it was stated that, by mass balance method and CT method, the purity of the analyte was determined, presenting an average of 99.80% with an extended uncertainty of 0.37% (*k* = 2). These methods, in addition to ensure validation of measurement methods, can also be used to improve the accuracy of measured data as well as control the quality of diosgenin in traditional herbs and pharmaceutical formulations [130].

### 3.2. Biological Matrices

Concerning biological matrices, up to date, to our knowledge, only two GC-MS with single-ion monitoring (SIM) mode methods have been reported to determine the content of diosgenin in the gastrointestinal tract of a lamb [131] and rats plasma [132]. Recently, the UPLC-QTOF-MS technique was applied in a study conducted in rat biosamples collected after oral administration of saponins from tree* Dioscorea* species as well as protodioscin (PD), pseudoprotodioscin (PSD), dioscin (DC), and diosgenin (DG). This study allowed comparing the metabolic profiles of these saponins and diosgenin as well as analyzing the levels of metabolites, by monitoring the chemical profiles of plasma, feces, and urine of rats during 36 hours. Interestingly, it was proven that diosgenin is one of the major metabolites found in plasma and feces (excluding urine) in all examined groups of rats [133]. Furthermore, the UPLC-UV/MS technique was applied to study the in vitro ADME properties of diosgenin and dioscin from* Dioscorea villosa*, including the stability analysis in biological fluids (gastric and intestinal fluids), intestinal absorption, and metabolic stability. Remarkably, it was evidenced that dioscin has better intestinal permeability than diosgenin and is converted to diosgenin in both gastric and intestinal fluids. No phase I metabolism was detected for both compounds and diosgenin probably undergoes phase II metabolism [14].

A LC-ESI-MS/MS method has also been developed and validated to determine the diosgenin levels in plasma from normal and hyperlipidemic rats. Single-ion monitoring (SIM) was used for quantification and the LOQ was 13 ng/mL. Sarsasapogenin was used as internal standard due to its structural similarity to diosgenin. The results showed an increase in the absorption of diosgenin in hyperlipidemic rats when compared with normal rats [134]. Later, Taketani et al. [87] developed a purification method for quantitative determination of diosgenin, dioscin, and protodioscin in plasma of fenugreek-fed mice, which consisted in deproteination of plasma samples, SPE with successive washes, and then analysis by LC-ESI-MS/MS. Moreover, the LC-MS technique was used for bioavailability studies of diosgenin in inclusion complexes with cyclodextrins in Caco-2 cell monolayers and rat jejunum. Interestingly, bioavailability of diosgenin in the presence of *β*-cyclodextrin derivatives was near 4- to 11-fold higher than that of diosgenin suspension [135]. The same research group evaluated, again by using LC-MS, the effect of diosgenin liquid crystals combined with cyclodextrin to increase the bioavailability of this steroid, after oral administration to rats [136].

## 4. Conclusions

Diosgenin, a steroid saponin which is found in a number of plant species, is reported to be a promising bioactive biomolecule with diverse important medicinal properties, including hypolipidemic, hypoglycaemic, antioxidant, anti-inflammatory, and antiproliferative activities.

For this reason, diosgenin is a potential molecule of interest in the prevention/treatment of several diseases. However, the determination of diosgenin specific targets is of major relevance to further validate its applications in the prevention and treatment of health conditions. The high potential of this compound, its analogues, or combinations of this compound with others is already proven; however, it is important to develop carrier systems, such as nanoparticles, to direct them to the place where diosgenin acts improving efficacy and reducing eventual side effects.

Due to its pharmacological relevance, several analytical assays have been reported in the literature over the last years to detect and quantify diosgenin in different matrices, including natural sources and pharmaceutical compositions containing it, and also in animal matrices in pharmacological studies. These assays involved spectrophotometry, gravimetry, classical TLC, and more recent advances of this technique (densitometric-TLC and HPTLC), ELISA, GC, LC, UPLC, UHPLC, and HPLC, coupled to different detectors, mainly UV, DAD, and MS. Of these, the HPLC method is probably the most used for the quantification of this compound. Most of these analytical methods have been validated to current standards and have been used successfully in different laboratories mainly to evaluate diosgenin levels in different plant sources. Despite the fact that, for example, GC-MS and HPLC-UV technologies are well-known and accessible in most laboratories nowadays and thus widely used, the use of LC-MS has clearly increased over the last few years and it is expected to continue to increase due to its advantages. As it is expected that instruments will become even more sensitive in the future, the use of highly sensitive and accurate mass spectrometers will become more widespread. Furthermore, as a consequence of the increased sensitivity of analytical equipment, there is also a tendency in reducing sample size, with clear advantages from the analytical point of view.

## Figures and Tables

**Table 1:** Analytical methodologies in different specimens for diosgenin determinations by means of HPLC and UHPLC.

Matrix	Sample amount	Extraction process	Chromatographic conditions	Instrumental analysis	Linear range	LOD/LOQ	References
*Dioscorea *species and related medicinal plants (*Smilax* and *Heterosmilax *species)	0.5 g	15 mL of methanol at room temperature for 0.5 h Hydrolysis with HCl 10% under vacuum at 60°C LLE (10 mL of chloroform)	Mobile phase: 0.1% formic acid in water (A) and 0.1% formic acid in acetonitrile (B) using an isocratic elution of 82% (B) in 0–10 min. Flow rate at 0.3 mL/min Column temperature: 40°C Stationary phase: Waters BEH C_18_ column (2.1 × 100 mm, 1.7 *μ*m)	HPLC-DAD at 203 nm UPLC-MicroToFQ (ESI+)	1–500 *μ*g mL^−1^	0.3/0.8 ng mL^−1^	[117]

Root extracts and polyherbal formulations containing *Smilax China*	10 g	SPE (Soxhlet apparatus with petroleum ether, chloroform, and methanol)	HPTLC Mobile phase: toluene : ethyl acetate (7 : 3% v/v) HPLC Mobile phase: acetonitrile : water 90 : 10 (% v/v)	HPTLC and HPLC with densitometry: 425 nm	2.0–10 *μ*g mL^−1^	0.7/2 *μ*g mL^−1^	[118]

Berries extracts and formulations containing *Solanum nigrum*	20 g	LLE with 20% of H_2_SO_4_ in 70% IPA and hexane for 8 h	Mobile phase: acetonitrile : water 92 : 08 (% v/v). Flow rate at 1.0 mL/min Column temperature: 25°C Stationary phase: C_18_ Thermo Hypersil column (250 mm × 4.6 mm, 5 *μ*m)	HPLC-DAD at 203 nm	1.0–60 *μ*g mL^−1^	0.33/1.0 *μ*g mL^−1^	[119]

Cultured cells of *Dioscorea zingiberensis*	0.1 g	20 mL of 95% ethanol, for 2 h. Hydrolysis with 20 mL H_2_SO_4_ 1 M at 121°C for 2 h. LLE with petroleum ether. The combined petroleum and NaOH 1 M.	Mobile phase: acetonitrile : water 90 : 10 (% v/v) Flow rate at 1.0 mL/min Column temperature: 30°C Stationary phase: reversed-phase Agilent TC-C_18_ column (250 × 4.6 mm, 5 *μ*m)	HPLC-DAD at 203 and 410 nm	0.0625–1.000 *μ*g	0.0372/0.1127 *μ*g	[93]

Cosmeceutical formulations	2.5 g	LLE with 10 mL of methanol mixed with 50% of tetrahydrofuran	Mobile phase: water : acetonitrile 15 : 85 (% v/v) Column temperature: room temperature Stationary phase: Phenomenex Luna-C_18_ column (150 × 4.6 mm, 5 *μ*m)	HPLC-DAD at 210 nm	50–1000 *μ*g mL^−1^	10/30 *μ*g mL^−1^	[120]

Seed extract of *Trigonella foenum graecum*	1 g	SPE (Soxhlet with water and ethanol mixture (1 : 1) for 72 h at 70°C). 80 mL of HCl 3 N for 1 h at 100°C. LLE with diethyl ether	Mobile phase: acetonitrile : water (10 : 90 v/v) gradient mode. Flow rate at 1.0 mL/min. Column temperature: 30°C Stationary phase: reversed-phase Symmetry C_8_ column (250 × 4.6 mm, 5 *μ*m)	HPTLC and HPLC-DAD at 205 nm	—	—	[100]

Pharmaceutical forms containing *Trigonella foenum graecum*	0.01 g	25 mL of methanol for 15 min	Mobile phase: acetonitrile : water 90 : 10 (% v/v). Flow rate of 1.0 mL/min Column temperature: room temperature. Stationary phase: Phenomenex RP-C_18_ column (150 × 4.6 mm, 5 *μ*m)	HPLC-UV at 203 nm	2.0–10.0 *μ*g mL^−1^	0.520/1.577 *μ*g mL^−1^	[121]

Polyherbal formulation containing *Tribulus terrestres *Linn.extract	1 g	90 mL HCl 3 N for 1 h 30 at 100°C LLE with 75 mL diethyl ether 75 mL	Mobile phase: methanol : water 15 : 85 (% v/v), gradient mode. Flow rate at 1.0 mL/min Column temperature: 30°C Stationary phase: Symmetry RP-C_18_ column (250 × 4.6 mm, 5 *μ*m)	HPLC-DAD at 205 nm	25.0–75.0 *μ*g mL^−1^	—	[122]

Rhizomes or tubers of various *Dioscorea *species and dietary supplements	0.5 g for solids and 1 mL for liquids	9 to 25 mL of methanol	Mobile phase: acetonitrile : water 75 : 25 (v/v%) containing 0.05% formic acid. Flow rate at 0.27 mL/min. Column temperature: 40°C Stationary phase: Acquity UPLC™ BEH Shield RP_18_ (100 × 2.1 mm, 1.7 *μ*m)	UHPLC-ELSD and DAD	15.0–550 *μ*g mL^−1^	5.0–12/10-25 *μ*g mL^−1^	[123]

## Abbreviations

Akt: Protein kinase B
APTT: Activated partial thromboplastin time
BSA: Bovine serum albumin
COX-2: Cyclooxygenase-2 protein
CRM: Candidate reference material
CT: Coulometric titration
DAD: Diode array detection
ELISA: Enzyme-linked immunosorbent assay
ELSD: Evaporative light scattering detection
GC: Gas chromatography
GPx: Glutathione peroxidase
HPLC: High performance liquid chromatography
HPTLC: High performance thin-layer chromatography
HSCCC: High-speed counter-current chromatography
hTERT: Human telomerase reverse transcriptase
ICAM-1: Intercellular adhesion molecule 1
IGF-1: Insulin growth factor 1
IKK*β*: Inhibitor of nuclear factor kappa B: kinase subunit beta
IL: Interleukin
IRS-1: Insulin receptor substrate
LC: Liquid chromatography
LLE: Liquid-liquid extraction
LLOQ: Lower limit of quantification
LOD: Limit of detection
LOQ: Limit of quantification
MAE: Microwave-assisted extraction
MALDI-TOF/MS: Matrix assisted laser desorption/ionization-time of flight-mass spectrometry
MAPK: Mitogen-activated protein kinases
MS: Mass spectrometry
NF-kB: Nuclear factor kappa B
PAD: Photodiode array detection
PARP: Poly-ADP-ribose polymerase
PCNA: Proliferating cell nuclear antigen
PPT: Protein precipitation procedure
PT: Prothrombin time
QTOF-MS: Quadrupole time of flight mass spectrometry
SFE: Supercritical fluid extraction
SIM: Single-ion monitoring
SOD: Superoxide dismutase
SPE: Solid phase extraction
STAT3: Signal transducer and activator of transcription
TLC: Thin-layer chromatography
TT: Thrombin time
UHPLC: Ultra high performance liquid chromatography
UPLC: Ultra-performance liquid chromatography
VCAM-1: Vascular cell adhesion protein 1
VEGF: Vascular endothelial growth factor.
